# Problematic technology use and sleep quality in young adulthood: novel insights from a nationally representative twin study

**DOI:** 10.1093/sleep/zsad038

**Published:** 2023-04-28

**Authors:** Juan J Madrid-Valero, Timothy Matthews, Nicola L Barclay, Candice L Odgers, Terrie E Moffitt, Avshalom Caspi, Louise Arseneault, Alice M Gregory

**Affiliations:** Department of Health Psychology, Faculty of Health Sciences, University of Alicante, Alicante, Spain; Social, Genetic and Developmental Psychiatry Centre, Institute of Psychiatry, Psychology and Neuroscience, King’s College London, London, UK; Centre for Statistics in Medicine, Nuffield Department of Orthopaedics, Rheumatology, and Musculoskeletal Sciences, University of Oxford, Oxford, UK; Department of Psychological Science, University of California, Irvine, Irvine, CA, USA; Social, Genetic and Developmental Psychiatry Centre, Institute of Psychiatry, Psychology and Neuroscience, King’s College London, London, UK; Departments of Psychology and Neuroscience, Psychiatry and Behavioral Sciences, and Institute for Genome Sciences and Policy, Duke University, Durham, NC, USA; Social, Genetic and Developmental Psychiatry Centre, Institute of Psychiatry, Psychology and Neuroscience, King’s College London, London, UK; Departments of Psychology and Neuroscience, Psychiatry and Behavioral Sciences, and Institute for Genome Sciences and Policy, Duke University, Durham, NC, USA; Social, Genetic and Developmental Psychiatry Centre, Institute of Psychiatry, Psychology and Neuroscience, King’s College London, London, UK; Department of Psychology, Goldsmiths, University of London, London, UK

**Keywords:** Genetics, technology, sleep quality, twins

## Abstract

**Study Objectives:**

Digital technology use is associated with poor sleep quality in adolescence and young adulthood although research findings have been mixed. No studies have addressed the association between the two using a genetically informative twin design which could extend our understanding of the etiology of this relationship. This study aimed to test: (1) the association between adolescents’ perceived problematic use of digital technology and poor sleep quality, (2) whether the association between problematic use of technology and poor sleep quality remains after controlling for familial factors, and (3) genetic and environmental influences on the association between problematic use of technology and poor sleep quality.

**Methods:**

Participants were 2232 study members (18-year-old twins) of the Environmental Risk (E-Risk) Longitudinal Twin Study. The sample was 48.9% male, 90% white, and 55.6% monozygotic. We conducted regression and twin difference analyses and fitted twin models.

**Results:**

Twin differences for problematic use of technology were associated with differences for poor sleep quality in the whole sample (*p* < 0.001; *B* = 0.15) and also when we limited the analyses to identical twins only (*p* < 0.001; *B* = 0.21). We observed a substantial genetic correlation between problematic use of technology and sleep quality (rA = 0.31), whereas the environmental correlation was lower (rE = 0.16).

**Conclusions:**

Adolescent reported problematic use of digital technology is associated with poor sleep quality—even after controlling for familial factors including genetic confounds. Our results suggest that the association between adolescents’ sleep and problematic digital technology use is not accounted for by shared genetic liability or familial factors but could reflect a causal association. This robust association needs to be examined in future research designed to test causal associations.

## Graphical abstract

**Graphical Abstract:**
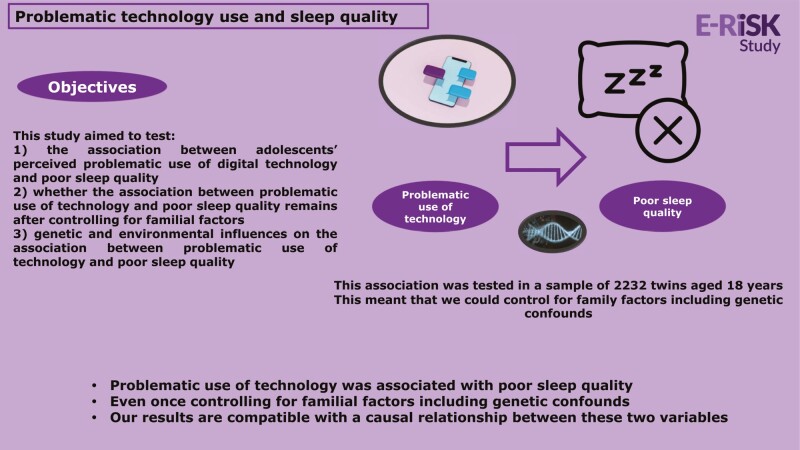

**Statement of Significance**

We live in a society with increased access to digital technology—and especially among young people. Sometimes, use of this technology can become problematic. This has the potential to impact sleep quality—although associations are likely to be complex and bidirectional. This study deepens our understanding of the association between technology use and sleep quality. Our results reveal a significant association between adolescents’ problematic digital technology use and sleep, even after controlling for familial factors. There was a genetic correlation between adolescents’ problematic use of digital technology and sleep quality. However, the association between the two variables was not fully explained by shared genetic risk or other familial factors, suggesting that there could be a causal relationship.

## Introduction

Digital technology use is associated with poor sleep quality in adolescence and young adulthood [1–3]. However, research findings have been mixed and recent studies among both children and adolescent that include relevant controls have shown that associations could be weaker than expected [4, 5]. In a recent study of adolescents, it was found that use of technology was associated with shorter sleep duration and later bedtime. However, most of the associations were nonsignificant when comparisons were made among individuals suggesting that a third factor that varies between adolescents could be underlying these associations [6]. In line with this, one possibility is that shared familial (genetic or shared environmental) factors influencing poor sleep quality may be shared with those for problematic technology use. For example, a lack of boundaries for children, could result in excessive technology use and inconsistent bedtimes, creating challenges for obtaining optimal sleep. Furthermore, parental control over technology use has been found to be cross-sectionally related to both time spent using technology and adolescent sleep (although longitudinal associations were not found) [7]. Despite a potential role for familial factors in the association between problematic use of technology and sleep quality, no studies have addressed this issue using a genetically informative twin design.

There are many reasons why the use of digital technology could be associated with sleep quality. For example, the former could lead to hyperarousal, which could interfere with sleep [8]. Additionally, devices such as mobile phones, tablets, computers, or video consoles can encourage continuous interactions which interfere with the necessary reduction in activity of the sympathetic nervous system and consequently hamper sleep onset [9, 10]. Furthermore, certain devices emit “blue light” which can disrupt the hormone of darkness—melatonin—and result in a missed cue that it is time to fall asleep. However, it is noteworthy that further research is needed to fully establish the nature of the relationship between “blue light,” melatonin, and sleep [11, 12]. Other devices can also emit sound which can disrupt sleep [13]. Conversely, those experiencing disrupted sleep have an increased opportunity to use technology.

The association between the use of technology and sleep disturbances has been examined in cross-sectional studies. For example, bedtime technology use has been associated with shorter sleep duration, poorer sleep quality, and sleep onset difficulties [14, 15]. A meta-analysis of the associations between portable screen media devices and sleep revealed that media device access and use at bedtime were associated with poor sleep quality and excessive daytime sleepiness [2]. Understanding these associations further is important as sleep disturbances have been related to several health problems including mental health difficulties (e.g. anxiety and depression) [16] and increased body mass index [17] as well as poor cognitive functioning [18, 19].

Longitudinal studies have also addressed the association between technology use and sleep in adolescents. For example, it has been found that greater time spent using technology predicts shorter subsequent sleep duration over a year period [20]. Furthermore, another longitudinal study (with measures at 11, 12, and 13 years of age) found that sleep duration and quality and technology use in early adolescence covaries [7]. One possibility is that familial factors (e.g. genetic and/or shared environmental factors) could be accounting for this association. However, despite the significant role of genetic factors on both technology use and sleep, no studies have addressed the association between the two variables while accounting for the role of genetic factors.

Therefore, this study aims to shed light on the complex relationship between the problematic use of technology and sleep quality by testing this association using a genetically informative design. We tested (1) the association between adolescents’ problematic use of technology and poor sleep quality controlling for covariates that could have impacted the results, specifically loneliness, neighborhood disorder, sex, socioeconomic status (SES), anxiety, depression, and mother’s insomnia, (2) whether the association between problematic use of technology and poor sleep quality remains after controlling for familial factors (including genetic confound), and (3) genetic and environmental influences on the association between problematic use of technology and sleep quality as well as certain covariates (i.e. loneliness, depression, and anxiety).

## Methods

### Participants

Participants were members of the Environmental Risk (E-Risk) Longitudinal Twin Study, which tracks the development of a birth cohort of 2232 British children. The sample was drawn from a larger birth register of twins born in England and Wales during 1994–1995 [21]. Full details about the sample are reported elsewhere [22]. Briefly, the E-Risk sample was constructed in 1999–2000, when 1116 families (93% of those eligible) with same-sex 5-year-old twins participated in home visit assessments. This sample comprised 56% monozygotic (MZ) and 44% dizygotic (DZ) twin pairs; sex was evenly distributed within zygosity (49% male). Ninety percent of participants were of white ethnicity.

Families were recruited to represent the UK population with newborns in the 1990s, to ensure adequate numbers of children in disadvantaged homes and to avoid an excess of twins born to well-educated women using assisted reproduction. The study sample represents the full range of socioeconomic conditions in Great Britain, as reflected in the families’ distribution on a neighborhood-level socioeconomic index (A Classification of Residential Neighborhoods [ACORN], developed by CACI Inc. for commercial use) [23, 24]. Specifically, E-Risk families’ ACORN distribution matches that of households nationwide: 25.6% of E-Risk families live in “wealthy achiever” ­neighborhoods compared to 25.3% nationwide; 5.3% versus 11.6% live in “urban prosperity” neighborhoods; 29.6% versus 26.9% live in “comfortably off” neighborhoods; 13.4% versus 13.9% live in “moderate means” neighborhoods, and 26.1% versus 20.7% live in “hard-pressed” neighborhoods. E-Risk underrepresents “urban prosperity” neighborhoods because such houses are likely to be childless.

Follow-up home visits were conducted when the children were aged 7 (98% participation), 10 (96%), 12 (96%), and 18 years (93%). There were 2066 individuals who participated in the E-Risk assessments at age 18, and the proportions of MZ (56%) and male same sex (47%) twins were almost identical to those found in the original sample at age 5. The average age of the twins at the time of the assessment was 18.4 years (SD = 0.36); all interviews were conducted after their 18th birthday. There were no differences between those who did and did not take part at age 18 in terms of SES assessed when the cohort was initially defined (*χ*^2^_(2, *N* = 2232)_ = 0.86, *p* = 0.65), age-5 IQ scores (*t*_(2208)_ = 0.98, *p* = 0.33), or age-5 emotional or behavioral problems (*t*_(2230)_ = 0.40, *p* = 0.69 and *t*_(2230)_ = 0.41, *p* = 0.68, respectively).

Home visits at ages 5, 7, 10, and 12 years included assessments with participants as well as their mothers (or primary caretaker). The home visit at age 18 included interviews only with the participants. The Joint South London and Maudsley and the Institute of Psychiatry Research Ethics Committee approved each phase of the study. Parents gave informed consent and twins gave assent between 5 and 12 years and then informed consent at age 18.

### Measures

#### Problematic digital technology use.

*Digital technology use-related impairments* were assessed based on adolescents’ perceptions of whether their digital technology use was problematic or impairing aspects of their daily life using an adapted version of the Compulsive Internet Use Scale [25]. The Compulsive Internet Use Scale was adapted for use in this study in two ways. First, the term “internet use” was replaced by “use of technology” and defined as the use of “the internet, email, social networking sites and tools, mobile phones, and text messaging” to reflect the changing nature of online activities and communication in the decade since the original scale was developed. Second, a reduced set of items (11 items coded as 0: “never,” 1: “sometimes,” or 2: “often”) were used to represent the following features of compulsive internet/technology usage: *loss of control* (e.g. Do you find it difficult to stop using technology, such as the internet or your mobile phone, once you start?), *withdrawal symptoms* (e.g. Do you feel restless, frustrated, or irritated when you cannot access the internet or check your mobile phone?), *coping or mood modification* (Do you use technology to escape from your sorrow or get relief from negative feelings?), *preoccupation* (Do you choose to spend more time online over going out with others?) and *intra- and inter-individual conflict* (Do you neglect your daily obligations (work, school, or family life) because you are using technology?).

#### Poor sleep quality.

At age 18 years, *sleep quality* over the past month was assessed using the Pittsburgh Sleep Quality Index (PSQI) [26], which is a questionnaire measure containing 18 items. Items include both open-ended questions (e.g. “During the past month, when have you usually gone to bed at night?”) and fixed-choice questions (“During the past month, how would you rate your sleep quality overall? ‘Very good; Fairly good; Fairly bad or Very bad’”). Questions tap a range of aspects of sleep quality and can be used to derive seven component scores (self-reported sleep quality, sleep latency, sleep duration, habitual sleep efficiency, sleep disturbances, use of sleep medications, and daytime dysfunction) as well as a global score. Higher scores on this measure reflect poorer sleep quality. The PSQI is reported to have internal consistency and test–retest reliability in the.8 range [26–28]. In the current sample, the mean was 5.39 (SD = 3.18), and the Cronbach’s alpha was.69. A substantial proportion (39.9%) of the participants scored >5 on the PSQI, which has been proposed as a clinical cutoff [26]. The PSQI score correlates highly with other measures of sleep such as sleep diary data [28].

#### Loneliness.

Loneliness was assessed when participants were 18 using four items from the University of California, Los Angeles (UCLA) Loneliness Scale, Version 3 [29]: “How often do you feel that you lack companionship?,” “How often do you feel left out?,” “How often do you feel isolated from others?,” and “How often do you feel alone?.” A very similar short form of the UCLA scale has previously been developed for use in large-scale surveys, and correlates strongly with the full 20-item version [30]. The scale was administered as part of a computer-based self-complete questionnaire. Interviewers were blind to participants’ responses. The items were rated “hardly ever” (0), “some of the time” (1), or “often” (2). Items were summed to produce a total loneliness score.

#### Neighborhood disorder.

Self-reports of neighborhood characteristics were collected via face-to-face interviews with participants at age 18. Neighborhood problems were measured by asking participants (6 items coded as 0: “not true,” 1: “sometimes true,” or 2: “often true”) if certain types of disorder were a problem in their area, such as “litter, broken glass, rubbish in public places” and “groups of young people hanging out and causing trouble” (coded 0–2). Items were summed to produce a scale of perceived neighborhood disorder.

#### Depression and anxiety symptoms.

Symptoms of major depressive disorder and generalized anxiety disorder at age 18 were assessed via a structured clinical interview, based on criteria in the Diagnostic and Statistical Manual of Mental Disorders, Fourth Edition [31]. The total number of symptoms (up to nine for depression and up to 6 for anxiety) were summed to create scales.

#### Mothers’ insomnia.

Participants’ mothers reported their symptoms of insomnia when participants were aged 12. A diagnosis of insomnia was made based largely on the criteria outlined by the Diagnostic and Statistical Manual of Mental Disorders, Fourth Edition. Specifically, mothers were asked if they experienced difficulty falling asleep, difficulty staying asleep, or problems waking too early. Answers were provided on a five-point scale (0 = none; 1 = mild; 2 = moderate; 3 = severe; and 4 = very severe). Mothers were also asked “how much do sleep problems interfere with your daily functioning?” (1 = not at all to 5 = very much). If mothers reported a sleep difficulty that they considered to be “severe” or “very severe” and reported an interference score ≥3, they were considered to have insomnia.

### Statistical analysis

To test the association between adolescents’ problematic use of technology and poor sleep quality, we conducted univariate and multivariate regressions adjusting for loneliness, neighborhood disorder, sex, SES, anxiety, depression, and mother’s insomnia when the participants were 12 years of age. These covariates were selected since they showed significant associations with sleep quality in this sample (*B* > 0.2 and *p* < 0.001). Covariates were assessed at 18 years except for the mother’s insomnia which was only measured when the twins were 12 years of age. We adjusted standard errors to account for the nonindependence of twin observations [32].

To test whether the association between problematic use of technology and poor sleep quality remains after controlling for familial factors, we used a twin difference method removing the intercept term as recommended elsewhere [33]. This involved computing a within twin-pair difference score by subtracting one twin’s problematic use of technology score from that of the cotwin and doing the same for their ratings of sleep quality. These difference scores represent variance explained by genetic differences and unique environmental exposures. Relative difference scores were also computed for the covariates. When the analyses are limited to MZ twins, difference scores represent variance accounted by unique environmental exposure only. Thus, if within-twin-pair differences in problematic use of technology correlate with within-pair differences in sleep quality for MZs, this association is explained by environmental factors that are unique to one twin.

Twin studies allow us to estimate the role of genetic and environmental factors on variance in a single variable or the covariance between multiple variables [34]. Making use of the difference between MZ twins (who share 100% of their DNA) and DZ twins (who share on average, 50% of their segregating DNA) the variance of one phenotype can be decomposed into genetic and environmental influences. Genetic influences can be divided into those that are additive (A; the sum of allelic effects across all loci) and nonadditive (D; the effects of genetic dominance). On the other hand, environmental contributions are shared (C; influences that make twin pairs raised in the same family similar to each other) and non-shared (E, effects that make family members less alike) [35].

Shared environmental factors and nonadditive genetic factors cannot be estimated at the same time using just data from twins reared together. Therefore, the selection of an ACE or ADE model is based on the pattern of MZ/DZ correlations. Typically, an ADE model is selected when the DZ correlation is lower than half of the MZ correlation. In contrast, an ACE model has been selected if the DZ correlation is greater than half of the MZ twin correlation [35, 36]. Univariate models were fitted and twin modeling assumptions were checked in these models.

We fitted a multivariate correlated factors model to estimate genetic and environmental contributions to both individual variance and sources of covariance. This model allows us to understand genetic and environmental influences on the association between problematic use of technology and poor sleep quality. In a multivariate model, the covariance decomposition allows us to examine the extent to which the phenotypic correlation between two traits is accounted for by genetic and environmental factors. The proportion explained by genetic factors is called bivariate heritability. Additionally, genetic and environmental correlations can be computed which reflect the extent to which the genetic/environmental factors underlying one trait overlap with the genetic/environmental factors that influence the other trait [37]. Sex was added as a covariate to the models. All variables were log+1 transformed to reduce skewness (variables ranged from 0.98 to 1.70 before transformation and from −0.40 to 1.43 after transformation). Sensitivity analyses were conducted on untransformed data (and heritability estimates changed by less than 3%).

## Results

Females reported poorer sleep quality than males (${\bar{x}}_{males}$= 5.07; ${\bar{x}}_{females}$ = 5.69; *p* < 0.001), more symptoms of depression (${\bar{x}}_{males}$= 1.44; ${\bar{x}}_{females}$= 2.13; *p* < 0.001) and symptoms of anxiety (${\bar{x}}_{males}$= 0.67; ${\bar{x}}_{females}$= 1.21; *p* < 0.001) (Table 1). All the covariates (higher levels of loneliness, depression, anxiety, neighborhood disorder, maternal insomnia, and female sex) were associated with poor sleep quality in both the univariate and multivariate models, except for SES which was not significantly associated with poor sleep quality in the multivariate model (Table 2).

**Table 1.** Descriptive statistics for the total sample and by sex at age 18 years

	Total sample (*N* = 2232)	Males (*N* = 1092)	Females (*N* = 1140)	*p*
		**M(SD)**		
Problematic use of technology (*N* = 2055)	4.54 (3.9)	4.45 (3.8)	4.62 (4.0)	0.367
Poor sleep quality (*N* = 2065)	5.39 (3.2)	5.07 (3.0)	5.69 (3.3)	**<0.001**
Loneliness (*N* = 2051)	1.57 (1.9)	1.51 (1.9)	1.62 (2.0)	0.223
Depression symptoms (*N* = 2063)	1.81 (3.0)	1.44 (2.7)	2.13 (3.2)	**<0.001**
Anxiety symptoms (*N* = 2060)	0.95 (1.8)	0.67 (1.5)	1.21 (2.0)	**<0.001**
Neighborhood disorder (*N* = 2062)	3.12 (3.0)	3.19 (3.0)	3.05 (3.0)	0.364

For all variables, a high score represents higher levels of symptoms.

*P*-value refers to significance levels for the differences between males and females.

**Table 2.** Univariate and multivariate regression analyses predicting poor sleep quality

		Univariate				Multivariate^*^		
	Coefficient	95% CI for the coefficient		*P*-value	Coefficient	*95% CI for the coefficient*		*P*-value
Problematic use of technology	0.23	0.18	0.28	**<0.001**	0.10	0.06	0.15	**<0.001**
Loneliness	0.29	0.24	0.34	**<0.001**	0.13	0.08	0.18	**<0.001**
Depression symptoms	0.37	0.32	0.42	**<0.001**	0.23	0.17	0.28	**<0.001**
Anxiety symptoms	0.28	0.23	0.33	**<0.001**	0.08	0.03	0.13	**0.002**
Neighborhood Disorder	0.20	0.15	0.25	**<0.001**	0.09	0.05	0.14	**<0.001**
Sex	0.20	0.10	0.29	**<0.001**	0.11	0.03	0.20	**0.009**
Maternal Insomnia	0.45	0.27	0.62	**<0.001**	0.26	0.11	0.42	**0.001**
SES^*^								
Low	0.23	0.11	0.35	**<0.001**	0.06	−0.04	0.18	0.224
Medium	0.07	−0.04	0.18	0.218	0.01	−0.09	0.10	0.924

For SES the medium and low groups are compared to the high SES group.

^*^The multivariate model predicts poor sleep quality from problematic use of technology (controlling for the other variables).

### Association between problematic use of technology and poor sleep quality

Problematic use of technology was associated with poor sleep quality (Table 2). This association remained significant, after controlling for loneliness, depression symptoms, anxiety symptoms, neighborhood disorder, sex, maternal insomnia, and SES. As the scale for problematic use of technology has one item referring to sleep “Are you short of sleep due to being on your phone or internet late” a sensitivity analysis testing the association between sleep quality and problematic use of technology was performed excluding this item from the original scale. Similar results were found (*B* = 0.23; *p* < 0.001 and *B* = 0.21; *p* < 0.001 for the original scale and for that excluding the sleep item respectively). We, therefore, present results from the full scale to ensure comparability with other studies. Of note, the associations between problematic use of technology and each component of the PSQI (controlling for covariates) are presented in Supplementary Tables 1–7. As associations were small and nonsignificant for some of the components, further analyses focus on the full sleep quality scale.

### Association between problematic use of technology and poor sleep quality controlling for familial factors

The unadjusted regression model showed that twin differences in problematic use of technology scores were associated with twin differences in sleep quality scores. This association remained significant when adjusting for the covariates. This association also remained significant and was not attenuated when limited to MZ twins (controlling for genetic and shared environmental factors). This indicates that problematic use of technology is associated with poor sleep quality via a nonfamilial environmental pathway (Table 3).

**Table 3.** Association between poor sleep quality and problematic use of technology controlling for familial factors—Regression twin difference design

MZ and DZ Twins (unadjusted model. *n* = 1012)	*Coefficient*	*95% CI for the coefficient*		*P*-value
Problematic use of technology	0.15	0.09	0.21	**<0.001**
MZ and DZ Twins (adjusted model. *n* = 993)	*Coefficient*	*95% CI for the coefficient*		*P*-value
Problematic use of technology	0.08	0.02	0.14	**0.014**
Loneliness	0.10	0.04	0.17	**0.002**
Depression symptoms	0.12	0.05	0.18	**<0.001**
Anxiety symptoms	0.15	0.08	0.21	**<0.001**
Neighborhood disorder	0.05	−0.01	0.11	0.118
MZ Twins (unadjusted model. *n* = 573)	*Coefficient*			*P*-value
Problematic use of technology	0.21	0.13	0.28	**<0.001**
MZ Twins (adjusted model. *n* = 564)	*Coefficient*	*95% CI for the coefficient*		*P*-value
Problematic use of technology	0.15	0.07	0.23	**<0.001**
Loneliness	0.09	0.00	0.17	**0.039**
Depression symptoms	0.06	−0.02	0.15	0.144
Anxiety symptoms	0.15	0.06	0.23	**0.001**
Neighborhood disorder	0.00	−0.08	0.08	0.999

All measures were treated as continuous variables. The cotwin analysis was performed using relative twin differences. Unadjusted models are those which do not include possible covariates. Adjusted models included the covariates loneliness, depression, anxiety, and neighborhood disorder.

### Genetic and environmental influences on the association between problematic use of technology and sleep quality

Univariate models were fitted for each phenotype included in the multivariate twin model (i.e. poor sleep quality, loneliness, problematic use of technology, depression, and anxiety). An ADE model was fitted for each phenotype as indicated by the pattern of correlations. For loneliness we also fitted an ACE model since the pattern of correlations could also suggest an ACE model. The best fit was provided by an AE model in all cases and all twin model assumptions were met (Supplementary Table 8). A multivariate model with five phenotypes (problematic use of technology, poor sleep quality, loneliness, depression, and anxiety) was also fitted, in order to disentangle the role of the genetic and environmental factors on the association between these phenotypes. MZ correlations were always higher than DZ correlations, indicating that genetic factors are involved (Table 4). The best fit was provided by an AE model (AIC_AE_ = 20318.45, AIC_ACE_ = 20327.87; AIC_ADE_ = 20327.87; Supplementary Table 9). Results from this model show that the five phenotypes are each moderately heritable (*A* = 0.33, 0.38, 0.35, 0.29, and 0.23 for sleep quality, loneliness, problematic use of technology, depression, and anxiety, respectively). A moderate genetic correlation (rA) was found between problematic use of technology and poor sleep quality (rA = 0.31) whereas the non-shared environmental correlation was lower (rE = 0.16). The association between problematic use of technology and poor sleep quality was explained roughly equally by genetic (49%) and environmental (51%) factors (Table 5). These proportions were calculated as follows: square root A(problematic use of technology) × Square root A(poor sleep quality) × rA for problematic use of technology and poor sleep quality. This is then divided by the phenotypic correlation (rPh) between problematic use of technology and poor sleep quality (i.e. ( =0.49). We also found significant genetic correlations between the rest of the variables and poor sleep quality (ranging from 0.39 for anxiety to 0.71 for depression) and non-shared environmental correlations (ranging from 0.15 for loneliness to 0.18 for anxiety).

**Table 4.** Twin correlations within and across traits

Monozygotic twins					
	Poor sleep quality	Loneliness	Problematic use of technology	Depression	Anxiety
Poor sleep quality	**0.33 (0.26,0.39)**				
Loneliness	0.20 (0.14,0.25)	**0.38 (0.32,0.45)**			
Problematic use of technology	0.10 (0.05,0.16)	0.16 (0.10,0.21)	**0.35 (0.28,0.42)**		
Depression symptoms	0.22 (0.17,0.27)	0.21 (0.15,0.26)	0.14 (0.09,0.19)	**0.29 (0.22,0.36)**	
Anxiety symptoms	0.11 (0.06,0.16)	0.22 (0.16,0.27)	0.08 (0.03,0.14)	0.24 (0.19,0.29)	**0.23 (0.16,0.30)**
Dizygotic twins					
Poor sleep quality	**0.16 (0.13,0.20)**				
Loneliness	0.10 (0.07,0.12)	**0.19 (0.16,0.22)**			
Problematic use of technology	0.05 (0.03,0.08)	0.08 (0.05,0.11)	**0.17 (0.14,0.21)**		
Depression symptoms	0.11 (0.08,0.14)	0.10 (0.08,0.13)	0.07 (0.04,0.10)	**0.15 (0.11,0.18)**	
Anxiety symptoms	0.05 (0.03,0.08)	0.11 (0.08,0.13)	0.04 (0.01,0.07)	0.12 (0.09,0.15)	**0.12 (0.08,0.15)**

Correlations were obtained from the multivariate AE model.

**Table 5.** Multivariate genetic AE model

	Poor sleep quality	Loneliness	Technology	Depression	Anxiety
Additive genetic and non-shared environmental overlap between phenotypes					
Poor sleep quality					
Loneliness	rA = 0.55 (0.42,0.69) rE = 0.15 (0.08,0.22) rPh = 0.29 (0.25,0.33)				
Problematic use of technology	rA = 0.31 (0.15,0.46) rE = 0.16 (0.09,0.23) rPh = 0.21 (0.17,0.25)	rA = 0.43 (0.30,0.56) rE = 0.15 (0.07,0.22) rPh = 0.25 (0.21,0.29)			
Depression symptoms	rA = 0.71 (0.56,0.86) rE = 0.16(0.09,0.23) rPh = 0.33 (0.29,0.37)	rA = 0.62 (0.49,0.74) rE = 0.26 (0.19,0.33) rPh = 0.38 (0.34,0.42)	rA = 0.44 (0.28,0.60) rE = 0.09 (0.02,0.17) rPh = 0.20 (0.16,0.25)		
Anxiety symptoms	rA = 0.39 (0.21,0.57) rE = 0.18 (0.11,0.25) rPh = 0.24 (0.19,0.28)	rA = 0.72 (0.57,0.89) rE = 0.19 (0.12,0.26) rPh = 0.35 (0.31,0.39)	rA = 0.29 (0.10,0.47) rE = 0.16 (0.09,0.23) rPh = 0.19 (0.15,0.24)	rA = 0.92 (0.76,1) rE = 0.24 (0.17,0.31) rPh = 0.42 (0.38,0.45)	
Additive genetic and non-shared environmental influences on the phenotypes and their associations					
Poor sleep quality	**A = 0.33 (0.26,0.39)** **E = 0.67 (0.61,0.74)**				
Loneliness	A = 0.67 (0.52,0.83) E = 0.33 (0.17,0.48)	**A = 0.38 (0.32,0.45)** **E = 0.62 (0.55,0.68)**			
Problematic use of technology	A = 0.49 (0.26,0.71) E = 0.51 (0.29,0.74)	A = 0.63 (0.44,0.81) E = 0.37 (0.19,0.56)	**A = 0.35 (0.28,0.42)** **E = 0.65 (0.58,0.72)**		
Depression symptoms	A = 0.67 (0.52,0.82) E = 0.33 (0.18,0.48)	A = 0.55 (0.42,0.67) E = 0.45 (0.33,0.58)	A = 0.69 (0.45,0.93) E = 0.31 (0.07,0.55)	**A = 0.29 (0.22,0.36)** **E = 0.71 (0.64,0.78)**	
Anxiety symptoms	A = 0.46 (0.25,0.67) E = 0.54 (0.33,0.75)	A = 0.62 (0.48,0.76) E = 0.38 (0.24,0.52)	A = 0.42 (0.15,0.67) E = 0.58 (0.33,0.85)	A = 0.58 (0.45,0.70) E = 0.42 (0.30,0.55)	**A = 0.23 (0.16,0.30)** **E = 0.77 (0.70,0.84)**

Bold figures in the lower part of the table represent within-trait standardized components of the variance and figures below the diagonal represent the standardized components of the covariance; A, additive genetic influence; E, non-shared environmental influence; rA, additive genetic correlation; rE, non-shared environmental correlation; rPh, phenotypic correlation from the model.

## Discussion

Our results suggest a robust association between adolescents’ perceived problematic use of digital technologies and poor sleep quality after controlling for familial factors. This association was of a small-moderate effect. Similar (or slightly higher) effect sizes were found for anxiety and depression which are two of the most studied predictors of poor sleep quality [38]. Recent studies have addressed the importance of identifying small effect sizes when it comes to psychological variables since they are influenced by multiple factors [39]. Furthermore, small statistical effects can have large societal-level effects [40].

The robust association reported here is consistent with previous studies which have revealed that greater use of technology is associated with poorer sleep quality and shorter sleep duration [1, 14, 15, 41]. In this study, we controlled for risk factors shared by twins as well as risk factors not shared by twins including loneliness, depression, anxiety, and neighborhood disorder. This approach allowed us to test the association between problematic use of technology and poor sleep quality with a high level of control. This suggests that a causal relationship between the variables is likely [42].

### Genetic and environmental influences on the association between problematic use of technology and poor sleep quality

Twin analyses examining the genetic and environmental influences on the traits and their associations found that around one-third of the differences in adolescents’ problematic media use were explained by genetic factors in this sample. The comparison with other studies is difficult since there is only a handful of studies that have addressed media use within twin studies. Nonetheless, a previous study estimated the heritability of problematic internet use in adolescents to be between 58%–66% [43]. Another study, using two samples, found that genetic influences accounted for 34%–60% of the variance in how often teens make voice calls and 50%–53% for how often they send text messages [44]. As for sleep quality, approximately one-third of the variance was explained by genetic factors. This estimate is in line with other studies and meta-analyses on this topic [45–48]. Previous research suggests that genetic and environmental influences on sleep quality are not moderated by age [46]. Furthermore, we found genetic and environmental correlations which suggest that some of the factors contributing to problematic use of technology could also contribute to poor sleep quality (e.g. genes affecting both phenotypes and certain environmental factors such as peer group technology use for example). Overall, this association was explained roughly equally by genetic and environmental factors which means that during adolescence both genetic and environmental factors play an important role in explaining the association between problematic use of technology and poor sleep quality.

#### Possible mechanisms.

Different mechanisms could underlie the association between problematic media use and poor sleep quality. As discussed, exposure to blue light during the evening hours could interfere with melatonin release, which could impact sleep [49]. Additionally, the use of technology near bedtime could have a negative impact on sleep quality via a stimulating effect on the brain [50, 51]. Using technology before bedtime can also delay the circadian clock [52] making an early bedtime more difficult to achieve. The use of electronic devices before sleeping could absorb time that could have been used to sleep. Additionally, screen-related activities such as playing certain types of video games could increase arousal or rumination can impact sleep quality. Finally, introducing technology into the bedroom is against standard sleep hygiene recommendations and could disrupt sleep via the emission of noise or light [53]. Conversely, people living with sleep problems could use more technology—exacerbating their sleep problems.

### Limitations

Our study should be interpreted in light of limitations. First, our analyses were cross-sectional meaning that we are not able to draw conclusions about the direction of effects between the variables. Nonetheless, the key association reported appeared to be robust—enduring controls for several measured and unmeasured covariates. Second, our measures of adolescents’ problematic use of technology and sleep quality were both self-report and additional objective measures of technology use and sleep quality would have been informative. Additionally, our measure of problematic use of technology is referred to as perceived problematic use which could be more strongly related to adolescent functioning as compared to actual usage [54]. Nonetheless, both questionnaires have been shown to be reliable (i.e. show adequate psychometric properties such as good test–retest reliability and internal consistency) [25, 27]. Researchers might want to consider including other types of measures in future research. For example, the use of technology can be active (e.g. playing video games) or passive (e.g. listening to music) and it is possible that the former type of use with greater interactivity could have a larger impact on sleep [51]. There has also been a recent increase in technology designed to support sleep (including phone apps incorporating mindfulness for example) and future work needs to establish the extent to which such technology could enhance, rather than hinder the sleep process. Future work may also benefit from measuring chronotype which could impact the relationship between use of technology and sleep quality. Eveningness as compared to a morning chronotype is more often associated with poorer sleep quality [55, 56]. People with an evening chronotype have a greater opportunity to use electronic devices late at night and this is one possible mechanism by which sleep could be impacted. A third limitation is that mothers’ insomnia was assessed when participants were aged 12 years, 6 years prior to the participants providing data on technology use and sleep quality. This covariate therefore may not accurately reflect the mother’s insomnia at the time, the participants reported difficulties with technology use and sleep. However, the mother’s insomnia was used as a proxy for genetic propensity for developing sleep problems making the timing less relevant. Furthermore, the father’s insomnia was not measured in this study so could not be included in these analyses. A fourth limitation of this study is that our results come from a sample that was mostly white ethnicity, comprising twins, and nonclinical participants. Therefore, results are not necessarily generalizable to other populations or clinical samples. To understand the association between problematic media use and sleep to a greater extent, our findings require replication in different samples.

## Conclusions

Adolescents reported problematic use of digital technology is associated with poor sleep quality—even after controlling for familial factors including genetic confounds. Our results suggest that the association between adolescents’ problematic digital technology use and sleep quality is not fully accounted for by shared genetic liability or familial factors but could reflect a causal association. Nonetheless, noncausal relationships can not be completely ruled out due to the cross-sectional nature of the study. Further research is needed to examine the various mechanisms by which problematic media use might result in poor quality sleep (or indeed the possibility that poor sleep quality is leading to problematic media use or that other factors are leading to both variables).

## Supplementary Material

zsad038_suppl_Supplementary_Tables

## Notes

The study was conducted at King’s College London, London, UK.
