# Supplementary material for: Problematic technology use and sleep quality in young adulthood: novel insights from a nationally representative twin study
Source: Sleep. 2023 Apr 28;46(6):zsad038. doi: 10.1093/sleep/zsad038 (PMC10262182; doi:10.1093/sleep/zsad038)
Supplement: zsad038_suppl_Supplementary_Tables [file zsad038_suppl_supplementary_tables.docx]

**Problematic technology use and sleep quality in young adulthood: Novel insights from a nationally representative twin study**

Juan J Madrid-Valero^1^; Timothy Matthews^2^; Nicola L Barclay^3^; Candice L Odgers^4^; Terrie E Moffitt^2,5^; Avshalom Caspi^2,5^; Louise Arseneault^2^; Alice M Gregory^6^

^1^Department of Health Psychology, Faculty of Health Sciences, University of Alicante, Alicante, Spain.

^2^Social, Genetic and Developmental Psychiatry Centre, Institute of Psychiatry, Psychology and Neuroscience, King's College London, London, United Kingdom.

^3^ Centre for Statistics in Medicine, Nuffield Department of Orthopaedics, Rheumatology, and Musculoskeletal Sciences, University of Oxford, Oxford, UK.

^4^Department of Psychological Science, University of California, Irvine, Irvine, CA, USA.

^5^Departments of Psychology and Neuroscience, Psychiatry and Behavioral Sciences, and Institute for Genome Sciences and Policy, Duke University, Durham, NC, USA.

^6^Department of Psychology, Goldsmiths, University of London, London, United Kingdom

**Corresponding authors:**

Juan J Madrid-Valero - Department of Health Psychology, Faculty of Health Sciences, University of Alicante, 03690 Alicante, Spain. Telephone: +34 965903400 ext. 1254 Email: [juanjose.madrid@ua.es](mailto:juanjose.madrid@ua.es)

Alice M. Gregory, Goldsmiths, University of London, New Cross, London, SE14 6NW.Telephone: +44 (0)20 7919 7959 Email: [a.gregory@gold.ac.uk](mailto:a.gregory@gold.ac.uk)

**Keywords:** Heritability, Sleep quality, Technology, Twins.

**Supplementary tables:**

- Supplementary Table 1. Multivariate logistic regression model (Outcome: subjective sleep quality)
- Supplementary Table 2. Multivariate logistic regression model (Outcome: sleep latency)
- Supplementary Table 3. Multivariate logistic regression model (Outcome: sleep duration)
- Supplementary Table 4. Multivariate logistic regression model (Outcome: habitual sleep efficiency)
- Supplementary Table 5. Multivariate logistic regression model (Outcome: sleep disturbances)
- Supplementary Table 6. Multivariate logistic regression model (Outcome: use of sleeping medication)
- Supplementary Table 7. Multivariate logistic regression model (Outcome: daytime dysfunction)
- Supplementary Table 8: Correlations, variance distribution and fitting statistics from univariate models
- Supplementary Table 9: Multivariate model fit statistics

Supplementary Table 1. Multivariate logistic regression model (Outcome: subjective sleep quality)

|  | Coefficient | p-value | *95% CI for the coefficient* | |
| --- | --- | --- | --- | --- |
| Problematic use of technology | 0.055 | **<0.001** | 0.030 | 0.080 |
| Loneliness | 0.127 | **<0.001** | 0.071 | 0.182 |
| Depression symptoms | 0.111 | **<0.001** | 0.072 | 0.150 |
| Anxiety symptoms | 0.037 | 0.210 | -0.021 | 0.094 |
| Neighborhood Disorder | 0.038 | **0.034** | 0.003 | 0.073 |
| Sex | -0.055 | 0.567 | -0.244 | 0.134 |
| Maternal Insomnia | 0.170 | 0.321 | -0.166 | 0.505 |
| SES  Low | 0.240 | **0.049** | 0.001 | 0.480 |
| Medium | -0.002 | 0.988 | -0.241 | 0.237 |

Note. For SES the medium and low groups are compared to the high SES group.

CI = confidence interval; SES = socioeconomic status.

Supplementary Table 2. Multivariate logistic regression model (Outcome: sleep latency)

|  | Coefficient | p-value | *95% CI for the coefficient* | |
| --- | --- | --- | --- | --- |
| Problematic use of technology | 0.020 | 0.078 | -0.002 | 0.041 |
| Loneliness | 0.090 | **<0.001** | 0.041 | 0.140 |
| Depression symptoms | 0.098 | **<0.001** | 0.064 | 0.132 |
| Anxiety symptoms | 0.060 | **0.016** | 0.011 | 0.109 |
| Neighborhood Disorder | 0.032 | **0.036** | 0.003 | 0.063 |
| Sex | 0.216 | **0.018** | 0.037 | 0.394 |
| Maternal Insomnia | 0.489 | **0.002** | 0.184 | 0.794 |
| SES  Low | 0.089 | 0.431 | -0.132 | 0.309 |
| Medium | -0.003 | 0.978 | -0.217 | 0.211 |

Note. For SES the medium and low groups are compared to the high SES group.

CI = confidence interval; SES = socioeconomic status.

Supplementary Table 3. Multivariate logistic regression model (Outcome: sleep duration)

|  | Coefficient | p-value | *95% CI for the coefficient* | |
| --- | --- | --- | --- | --- |
| Problematic use of technology | 0.026 | **0.029** | 0.002 | 0.050 |
| Loneliness | 0.077 | **0.005** | 0.023 | 0.132 |
| Depression symptoms | 0.068 | **<0.001** | 0.033 | 0.104 |
| Anxiety symptoms | 0.004 | 0.876 | -0.051 | 0.060 |
| Neighborhood Disorder | 0.045 | **0.005** | 0.014 | 0.077 |
| Sex | -0.108 | 0.265 | -0.298 | 0.082 |
| Maternal Insomnia | 0.253 | 0.103 | -0.051 | 0.559 |
| SES  Low | 0.289 | **0.020** | 0.046 | 0.532 |
| Medium | 0.176 | 0.134 | -0.544 | 0.407 |

Note. For SES the medium and low groups are compared to the high SES group.

CI = confidence interval; SES = socioeconomic status.

Supplementary Table 4. Multivariate logistic regression model (Outcome: habitual sleep efficiency)

|  | Coefficient | p-value | *95% CI for the coefficient* | |
| --- | --- | --- | --- | --- |
| Problematic use of technology | -0.004 | 0.747 | -0.032 | 0.023 |
| Loneliness | -0.005 | 0.853 | -0.059 | 0.049 |
| Depression symptoms | 0.069 | **<0.001** | 0.033 | 0.105 |
| Anxiety symptoms | 0.062 | **0.040** | 0.003 | 0.121 |
| Neighborhood Disorder | 0.025 | 0.123 | -0.007 | 0.057 |
| Sex | 0.300 | **0.003** | 0.105 | 0.494 |
| Maternal Insomnia | 0.294 | 0.071 | -0.025 | 0.614 |
| SES  Low | 0.263 | **0.035** | 0.019 | 0.508 |
| Medium | 0.042 | 0.729 | -0.195 | 0.279 |

Note. For SES the medium and low groups are compared to the high SES group.

CI = confidence interval; SES = socioeconomic status.

Supplementary Table 5. Multivariate logistic regression model (Outcome: sleep disturbances)

|  | Coefficient | p-value | *95% CI for the coefficient* | |
| --- | --- | --- | --- | --- |
| Problematic use of technology | 0.056 | **<0.001** | 0.026 | 0.086 |
| Loneliness | 0.021 | 0.479 | -0.037 | 0.079 |
| Depression symptoms | 0.120 | **<0.001** | 0.075 | 0.165 |
| Anxiety symptoms | 0.102 | **0.004** | 0.033 | 0.171 |
| Neighborhood Disorder | 0.104 | **<0.001** | 0.062 | 0.146 |
| Sex | 0.657 | **<0.001** | 0.424 | 0.889 |
| Maternal Insomnia | 0.561 | **0.012** | 0.123 | 1.000 |
| SES  Low | -0.010 | 0.946 | -0.296 | 0.276 |
| Medium | 0.001 | 0.999 | -0.276 | 0.276 |

Note. For SES the medium and low groups are compared to the high SES group.

CI = confidence interval; SES = socioeconomic status.

Supplementary Table 6. Multivariate logistic regression model (Outcome: use of sleeping medication)

|  | Coefficient | p-value | *95% CI for the coefficient* | |
| --- | --- | --- | --- | --- |
| Problematic use of technology | -0.007 | 0.838 | -0.075 | 0.061 |
| Loneliness | 0.051 | 0.438 | -0.078 | 0.180 |
| Depression symptoms | 0.199 | **<0.001** | 0.102 | 0.297 |
| Anxiety symptoms | 0.193 | **0.006** | 0.055 | 0.330 |
| Neighborhood Disorder | -0.025 | 0.532 | -0.105 | 0.054 |
| Sex | 0.171 | 0.534 | -0.369 | 0.711 |
| Maternal Insomnia | 0.860 | **0.026** | 0.103 | 1.616 |
| SES  Low | -0.203 | 0.585 | -0.931 | 0.525 |
| Medium | 0.028 | 0.936 | -0.653 | 0.709 |

Note. For SES the medium and low groups are compared to the high SES group.

CI = confidence interval; SES = socioeconomic status.

Supplementary Table 7. Multivariate logistic regression model (Outcome: daytime dysfunction)

|  | Coefficient | p-value | *95% CI for the coefficient* | |
| --- | --- | --- | --- | --- |
| Problematic use of technology | 0.097 | **<0.001** | 0.072 | 0.123 |
| Loneliness | 0.227 | **<0.001** | 0.171 | 0.284 |
| Depression symptoms | 0.100 | **<0.001** | 0.064 | 0.136 |
| Anxiety symptoms | 0.076 | **0.010** | 0.018 | 0.135 |
| Neighborhood Disorder | 0.039 | **0.030** | 0.004 | 0.073 |
| Sex | 0.071 | 0.449 | -0.112 | 0.255 |
| Maternal Insomnia | 0.241 | 0.179 | -0.110 | 0.592 |
| SES  Low | -0.491 | **<0.001** | -0.726 | -0.255 |
| Medium | -0.350 | **0.003** | -0.578 | -0.121 |

Note. For SES the medium and low groups are compared to the high SES group.

CI = confidence interval; SES = socioeconomic status.

Supplementary Table 8: Correlations, variance distribution and fitting statistics from univariate models

|  | **Model** | **Model for**  **comparison** | **A (95% CI)** | **C/D (95% CI)** | **E (95% CI)** | **df** | **-2LL** | **AIC** | **Diff-2LL** | **Diffdf** | **P** | **rMZ** | **rDZ** |
| --- | --- | --- | --- | --- | --- | --- | --- | --- | --- | --- | --- | --- | --- |
| Poor Sleep Quality |  |  |  |  |  |  |  |  |  |  |  | 0.34 (0.27, 0.41) | 0.12 (0.02, 0.21) |
|  | ADE |  | 0.12 (-0.26,0.48) | 0.23 (-0.16,0.63) | 0.66 (0.59,0.73) | 2060 | 3000.37 | 3010.37 |  |  |  |  |  |
|  | **AE** | ADE | **0.33 (0.26,0.40)** | **/** | **0.67 (0.60,0.74)** | **2061** | **3001.72** | **3009.72** | **1.35** | **1** | **0.25** |  |  |
|  | E | AE | **/** | **/** | 1 (1,1) | 2062 | 3078.92 | 3084.92 | 77.21 | 1 | <0.001 |  |  |
| Problematic use of echnology |  |  |  |  |  |  |  |  |  |  |  | 0.37 (0.29, 0.43) | 0.10 (0.01, 0.20) |
|  | ADE |  | 0.04 (-0.34,0.40) | 0.33 (-0.05,0.73) | 0.63 (0.56,0.70) | 2050 | 4839.07 | 4849.07 |  |  |  |  |  |
|  | **AE** | ADE | **0.35 (0.28, 0.41)** | **/** | **0.65 (0.59,0.72)** | **2051** | **4841.99** | **4849.99** | **2.92** | **1** | **0.09** |  |  |
|  | E | AE | **/** | **/** | 1 (1,1) | 2052 | 4925.01 | 4931.01 | 83.02 | 1 | <0.001 |  |  |
| Loneliness |  |  |  |  |  |  |  |  |  |  |  | 0.38 (0.31, 0.45) | 0.19 (0.09, 0.28) |
|  | ACE |  | 0.39 (0.16,0.61) | 0 (-0.20,0.18) | 0.62 (0.55,0.69) | 2046 | 4249.36 | 4259.36 |  |  |  |  |  |
|  | ADE |  | 0.38 (0,0.73) | 0.01 (-0.37,0.40) | 0.62 (0.55,0.69) | 2046 | 4249.36 | 4259.36 |  |  |  |  |  |
|  | **AE** | ADE | **0.38 (0.32,0.44)** | **/** | **0.62 (0.56,0.68)** | **2047** | **4249.36** | **4257.36** | **<0.001** | **1** | **0.97** |  |  |
|  | E | AE |  |  | 1 (1,1) | 2048 | 4355.02 | 4361.02 | 105.66 | 1 | <0.001 |  |  |
| Depression symptoms |  |  |  |  |  |  |  |  |  |  |  | 0.32 (0.25, 0.39) | 0.07 (0.00,0.16) |
|  | ADE |  | -0.04 (-0.43,0.34) | 0.36 (-0.04,0.76) | 0.68 (0.61,0.76) | 2058 | 5260.43 | 5270.43 |  |  |  |  |  |
|  | **AE** | ADE | **0.29 (0.22,0.36)** | **/** | **0.71 (0.64,0.78)** | **2059** | **5263.52** | **5271.52** | **3.09** | **1** | **0.08** |  |  |
|  | E | AE | / | / | 1 (1,1) | 2060 | 5324.06 | 5330.06 | 60.54 | 1 | <0.001 |  |  |
| Anxiety symptoms |  |  |  |  |  |  |  |  |  |  |  | 0.25 (0.18,0.33) | 0.07 (0.00,0.16) |
|  | ADE |  | 0 (-0.38, 0.36) | 0.26 (-0.13, 0.66) | 0.74 (0.67, 0.82) | 2055 | 4173.28 | 4183.28 |  |  |  |  |  |
|  | **AE** | ADE | **0.24 (0.16, 0.31)** | **/** | **0.76 (0.69,0.84)** | **2056** | **4175.00** | **4183.00** | **1.72** | **1** | **0.19** |  |  |
|  | E | AE | **/** | **/** | 1 (1,1) | 2057 | 4213.10 | 4219.10 | 38.10 | 1 | <0.001 |  |  |

A, additive genetic influence; C, shared environmental influence; D, dominant genetic influence E, non-shared environmental influence; -2LL, negative 2 log-likelihood; AIC, Akaike’s information criterion; CI, confidence interval; df, degrees of freedom; P-value, significance value of the likelihood-ratio chi-square test; rDZ, dizygotic correlations; rMZ, monozygotic correlations. Bold text indicates best fitting models

Supplementary Table 9: Multivariate model fit statistics

| Model | -2LL | df | Parameters | AIC | Diff-2LL | diffdf | p |
| --- | --- | --- | --- | --- | --- | --- | --- |
| ACE | 20217.87 | 10239 | 55 | 20327.87 |  |  |  |
| ADE | 20217.87 | 10239 | 55 | 20327.87 |  |  |  |
| AE | 20238.45 | 10254 | 40 | 20318.45 | 20.58 | 15 | .151 |
| E | 20548.39 | 10269 | 25 | 20598.39 | 309.94 | 15 | <.001 |

Note: -2LL, negative 2 log-likelihood; AIC, Akaike’s information criterion; df, degrees of freedom; p-value, significance value of the likelihood-ratio chi-square test
